# Domestic European Rabbits *Oryctolagus cuniculus*: A Super-Highway for the Spread of Emergent Viral Diseases to Other Lagomorphs?

**DOI:** 10.1155/tbed/1129135

**Published:** 2025-06-06

**Authors:** Elena Angulo, Juan Bárcena, Brian Cooke, Ramón C. Soriguer

**Affiliations:** ^1^Doñana Biological Station, EBD-CSIC, Calle Américo Vespucio, 26, Seville 41092, Spain; ^2^Animal Health Research Center (CISA-INIA/CSIC), Valdeolmos 28130, Madrid, Spain; ^3^Foundation for Rabbit Free Australia, P.O. Box 145, Collinswood SA 5081, Adelaide, Australia

**Keywords:** hepatitis E virus (HEV), meat, myxoma virus, rabbit distribution, rabbit haemorrhagic disease virus, RHDV (GI.1), RHDV2 (GI.2), trade

## Abstract

We propose that the worldwide spread of several viral diseases in European rabbits (*Oryctolagus cuniculus*) is facilitated by domestic rabbit meat production and associated international trade. This view is based on published records of the transfer of rabbit haemorrhagic disease viruses (RHDV/RHDV2) between countries and supported by data from the Food and Agriculture Organization (FAO) and the World Animal Health Information System (WAHIS) correlating the amount of rabbit meat produced and the number of rabbit haemorrhagic disease (RHD) outbreaks reported. Although RHDV was mainly confined to European rabbits, outbreak reporting rose after RDHV2 emerged and spread into many other lagomorph species. More than 80 species of native lagomorphs are now at risk from the disease in countries reporting RHD outbreaks. Our findings have implications for the maintenance of both industrial-scale cuniculture and village-scale production to combat poverty, for the future use of viruses for the biological control of pest rabbits and the conservation of native lagomorphs. Greater awareness of the risks of virus transfer in both directions between domestic rabbits and wild lagomorphs is important for future management of domestic rabbits and the conservation of native lagomorphs.

## 1. Introduction

In a wide-ranging review, Espinosa et al. [1] discussed the ways in which worldwide meat production increases the risk of epidemics. They considered that traditional food systems such as bushmeat and backyard farming increase the risks of disease transmission from wild animals. Furthermore, intensive meat production amplifies disease because of the high density of livestock, genetic relatedness, increased immunodeficiency, antibiotic use and the transport of farmed animals and their products.

Such risks are not confined to the major livestock industries, such as beef and chicken production. Using domestic European rabbits (*Oryctolagus cuniculus*) for meat production appears to be equally problematic. The recent successive emergences and worldwide spread of two different genotypes of rabbit haemorrhagic disease virus, RHDV and RHDV2, also referred to as *Lagovirus europaeus* GI.1 and GI.2 [2] are well documented and provide useful case studies for exploring the epidemic risk associated with rabbit meat production.

Both RHDV and RHDV2 are highly virulent Lagoviruses in the Caliciviridae family that can cause high mortality of 70%–95% in susceptible naive rabbits [3, 4]. These viruses appear to have arisen separately in farmed rabbits in Europe because both are closely related to non-pathogenic lagoviruses (RCVs) that circulate in both domestic and wild rabbits [5, 6]. Indeed, RHDV2 is a recombinant of RCV and an unknown virus [7, 8] reducing the likelihood of other potential origins, such as a jump from another species, as suggested by Merchan et al. [9].

RHDV was first described in China in domestic rabbits recently imported from East Germany [10]. Rabbit haemorrhagic disease (RHD) subsequently killed 140 million farmed domestic rabbits in China before adequate vaccines were developed [3]. From China it was spread on imported rabbit fur to South Korea [11] and to Mexico and Reunion in imported rabbit meat [12, 13]. Meanwhile, the spread of RHDV in Europe was also linked to trade in rabbit products. Cancellotti and Renzi [14] noted that Italy was importing 14,000 tonnes of fresh and frozen rabbit meat from Central European countries and another 500 tonnes of frozen meat from China annually when RHD first broke out in 1986. The virus spread through most of Europe by 1988 [13] but only reached Britain, a country which produces relatively little meat from domestic rabbits, in 1991 [15]. There it was imported in exhibition rabbits before infecting domestic rabbits and spreading through the wild rabbit population [16].

Nonetheless, not all transfers of the virus were inadvertent or associated with commercial rabbit production and stud rabbits. In 1995, RHDV was also deliberately released in Australia for the biological control of introduced pest rabbits [17] and subsequently illegally introduced into New Zealand by landholders for the same purpose [18]. The disease was also introduced into the Canary Islands by hunters restocking reserves with wild rabbits from mainland Spain [19].

RHDV2 was first detected among farmed rabbits in France in 2010 [4, 20] and soon spread through Europe in both domestic and wild rabbits. Its international spread was again mostly associated with domestic rabbit production and trade. For example, Ambagala et al. [21] describe the spread of the disease in domestic rabbits in small family farms through Cote d'Ivoire, Ghana and Nigeria in West Africa. It initially spread from port to port rather than overland, eventually reaching South Africa in 2022 [22]. It was noted that many people in Nigeria whose rabbits died from RHDV2 had recently introduced new breeding stock [23].

Something similar applied in North Africa. There was a progressive spread through rabbit farms in Morocco and Tunisia as RHDV2 replaced RHDV [24, 25]. RHDV2 was first recorded in Morocco in 2017 but could have arrived earlier given Morocco's proximity to Spain and Portugal, and it was the main virus circulating in rabbit farms in Tunisia and Algeria in 2018 [25, 26]. RHDV2 was also detected in Egypt in 2018 but remained confined to domestic rabbits in the governorates around Cairo until at least 2022, whereas disease outbreaks in domestic rabbits in Upper Egypt were caused by RHDV [27].

Thus, there is a strong initial case that both forms of RHDV originated in intensively farmed European rabbits and were spread, mainly by trade in rabbits and rabbit products, just as Espinosa et al. [1] thought was the case for disease spread in the major meat-producing livestock industries.

Of additional concern, RHDV2 proved to be highly contagious and unlike RHDV which was largely confined to European rabbits, it was shown to fatally infect other species of lagomorphs (Table 1) including Italian hares (*Lepus corsicanus*), Irish hares (*Lepus timidus hibernicus*), Sardinian Cape hares (*Lepus capensis mediterraneus*) and European brown hares (*Lepus europaeus*) [28, 29, 32, 34]. In North America, the accidental introduction of RHDV from China had been confined to Mexico and had been stamped out [12], and only occasional cases of RHDV had been noted in the USA, Canada and Cuba [43, 44]. By contrast, RHDV2 suddenly spread not only in domestic rabbits but also among jack rabbits (*Lepus* spp.) and cottontails (*Sylvilagus* spp.) in western USA and northern Mexico [38]. It also appeared in native lagomorphs in South Africa in the Western Cape region as well as in domestic rabbits there and around Johannesburg [22]. Included among the newly infected lagomorph species were threatened species such as pygmy rabbits (*Brachylagus idahoensis*) in North America [42] and the critically endangered riverine rabbit (*Bunolagus monticularis*) and red rock rabbits (*Pronolagus* spp.) in South Africa [45].

RHDV was considered a major threat to wild rabbits in Spain, Portugal and southern France, where rabbits are native animals and important key-stone species in Mediterranean ecosystems, modifying vegetation and supporting rare predator populations such as the Iberian lynx and Bonelli's Eagle [46, 47]. It was also considered a threat for the large populations of wild rabbits in islands such as Sicily and Sardinia. The rapid increase in the host range of RHDV2 greatly extended the problem, turning RHDV2 into a serious conservation issue on a worldwide scale [22].

In this paper, we predict from the published reports on the international spread of RHDV/RHDV2, that outbreaks of the disease should be strongly associated with areas where rabbit production is most intensive. To test this, we analyse reports of disease outbreaks in relation to data on worldwide rabbit production, and we confirm that there is a significant correlation between reporting of outbreaks of RHDV/RHDV2 and rabbit meat production.

We also list the numbers of native lagomorph species in countries where RHDV has been reported to highlight the potential risk of transfer of new viruses between domestic rabbits and wild lagomorphs in either direction. We then briefly consider other common viruses that cause diseases in European rabbits. Using well-known examples such as myxoma virus (MYXV) and rabbit hepatitis E virus (rHEV) and newly discovered viruses, we explore the potential of those viruses to spread like RHDV2 between different species of lagomorphs in the future. We then discuss the implications of the emergence of RHDV2 in terms of its effects on worldwide rabbit production, the further use of the virus for the biological control of rabbits, and the implications for the health and conservation of native lagomorph species. Finally, we briefly suggest some ideas for better managing rabbit diseases internationally.

## 2. Materials and Methods

### 2.1. Worldwide Domestic Rabbit Production and Relationship With RHD Outbreaks

To further substantiate that the spread of RHDV and RHDV2 was associated with domestic rabbit meat production globally, we compared reports of RHD outbreaks and data for rabbit meat production country by country. If we found no correlation, it would conclusively show that the spread of the disease was not closely linked to rabbit meat production, but a significant correlation would leave open or increase the likelihood that disease spread was facilitated by trade in rabbit products as published reports indicated.

Nonetheless, we did not anticipate a close relationship because reporting is often limited to first reports in countries previously free of the disease or if major changes in mortality caused by the disease are noted. Many countries no longer consider the diseases notifiable. For countries such as Nigeria, there were few reports of outbreaks despite the devastation caused to domestic rabbit production by the spread of RHDV2 [48]. According to Bello et al. [23] fewer than 25% of the farmers who lost rabbits as RHDV2 spread through Nigeria reported their losses to veterinary officials, resulting in serious under-estimation of the extent of disease spread. By contrast, New Zealand as an island nation with high quarantine standards and an interest in RHDV as a biological control agent for introduced pest rabbits headed the list of countries reporting RHDV and RHDV2 outbreaks.

Official figures on rabbit meat production by individual countries also underestimate the true scale of rabbit keeping because non-commercial production of rabbits for meat and fur is largely unregulated, and the numbers of rabbits kept as pets are unknown. In the USA, for example, rabbit meat production is relatively low, but there is an enormous pet rabbit industry estimated to be worth over USD 2 billion annually, which involves about 7 million domestic rabbits in almost 3 million households [49].

Data on reported outbreaks of RHDV and RHDV2 were obtained from the WAHIS portal of the World Organisation for Animal Health (WOAH), using the animal health data dashboard (https://wahis.woah.org/#/dashboards/country-or-disease-dashboard). The *Disease* selected was “rabbit haemorrhagic disease”; the *Animal type* was “terrestrial”; the *global status* was “present”; we selected “all animal” category which encompasses domestic and wild; and “all” for the variables *World regions* and *Countries*, *year and semester*. Information for outbreaks was only available from 2005 to 2024, but, because some countries had not reported the presence of RHD beyond 2022, we could not consider the two last years (2023–2024). This filtering selection gave 1185 events (Supporting information Table S1).

We also retrieved data from the Food and Agriculture Organization of the United Nations (FAO) using the FAOSTAT website (https://www.fao.org/faostat/en/#data/QCL) with the following terms: *item or domain* “Livestock primary” then “Meat of rabbit and hares, fresh or chilled”; we selected the *Unit* “t” (tonnes) and the *Element* “Production quantity”; then we selected in the *Flag description* “Official figure”; and finally, we selected all *Countries* and all *Years*. All years included the period from 1961 to 2022. This filtering selection gave 1066 data points (Supporting information Table S2).

We then asked whether the number of reported outbreaks depended on rabbit production in each country, considering two time periods. The first from 2005 until 2010, included the period when only RHDV was present. The second period covered the period 2010–2022 after RHDV2 had largely displaced RHDV worldwide (although the information on the virus genotype was not always available). The number of outbreaks reported by each country was summed, and average annual rabbit production for each country was calculated for each of these periods. We ran a general linear model in which the number of outbreaks was the dependent variable, which was log transformed (log + 1) and modelled with a Gaussian distribution and an identity link; the independent variables were the period (2005–2010 and 2010–2024) and the average of the yearly rabbit meat production for each country (in each period, log transformed, log + 1).

Because we were also interested in the relationship between the number of outbreaks and rabbit production independently of time, we removed the variable “period” and we ran the same model with only the average rabbit production for each country for the years 2005–2022, when the outbreaks were reported.

We also asked whether rabbit production differed between those countries that reported RHDV outbreaks and those which did not, considering the period when the outbreak occurred. We ran a general linear model in which rabbit production was the dependent variable, and the independent factors were the presence of RHD outbreaks (yes or no) and the time periods (2005–2010 and 2010–2024). The dependent variable, rabbit production, was log transformed (log+1) to use a Gaussian distribution with an identity link.

Finally, we asked whether rabbit production before the emergence of RHDV might influence whether countries would report outbreaks or the number of outbreaks. We used the data on rabbit production before the emergence of RHDV, from 1961 to 1985. We ran two models like the ones described above asking whether the total number of outbreaks (2005–2022) was correlated with rabbit production between 1961 and 1985 and then asked whether rabbit production differed between those countries that subsequently reported outbreaks and those that did not.

### 2.2. Risk of Transfer of New Viral Diseases to or From Native Lagomorphs

To evaluate the risk of a new viral disease spreading between domestic rabbits and wild native lagomorphs, we considered the frequency of reports from the countries where RHDV and RHDV2 occur and the number of native lagomorph species present in each country.

For each country that reported RHDV or RHDV2 outbreaks, the number of naturally occurring native and non-native (domestic, introduced, invasive) lagomorph species was obtained from two different information sources. First, we used Internet sources including the Global Biodiversity Information Facility (GBIF) [50], and the International Union for Nature Conservation (IUCN) summaries of local faunas. In the Red List of the IUCN (https://www.iucnredlist.org) [51] each lagomorph species was assessed for its distribution. Second, we searched in two books: the *Handbook of the Mammals of the World* [52] and the *Rabbits, hares and pikas: status and conservation action plan* [53, 54]. The final list of Lagomorph species for each country after removing synonyms is provided in Supporting Information Tables S3 and S4. We did not consider subspecies, as for many of them their status is not well-defined.

Lagomorph species were categorised as being native or non-native, and the native ones were further categorised according to their population trends and conservation status as reported by IUCN, and we calculated the percentage of lagomorph species with a decreasing trend and the percentage of lagomorph species whose conservation status was critically endangered (CR), endangered (EN) or vulnerable (VU).

Finally, because of the recent spread of RHDV2 into many species of wild lagomorphs we asked whether reporting of outbreaks in wild lagomorph populations had increased over time. We ran two generalised linear models, one for outbreaks in domestic rabbits and the other for the outbreaks in wild rabbits, in which the number of outbreaks for each year was the dependent variable (with a Poisson distribution with log link function) and the independent variable was the year in which the outbreaks were reported. Then, we calculated the percentage of outbreak reports in domestic vs. wild rabbits per year, and we tested the differences in this percentage between before and after 2010 using a generalised linear model (binomial distribution with logit link function. The dependent variable was the number of outbreaks in domestic rabbits with respect to the total number of outbreaks reported each year. The independent variable was the period (2005–2010 vs. 2011–2022). We used Proc Genmod (SAS v. 9.4; [55]) to run both analyses.

## 3. Results

### 3.1. Worldwide Domestic Rabbit Production and Relationship With Virus Outbreaks

**Figure 1:**
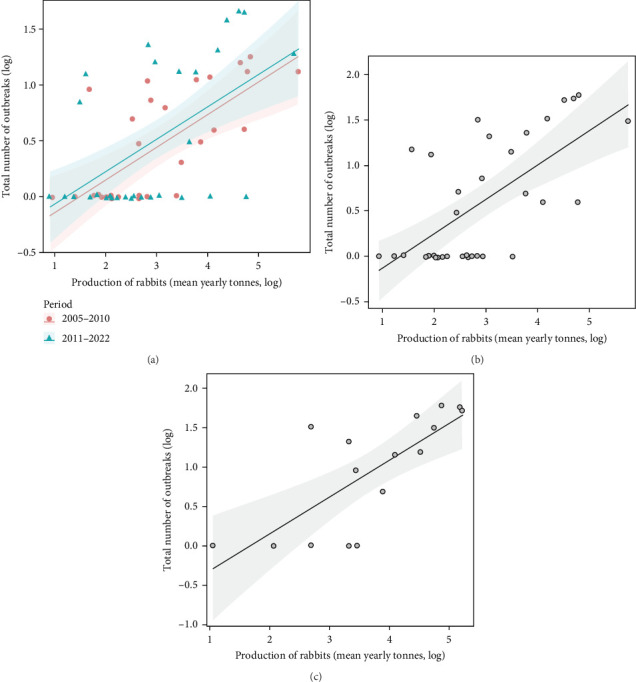
Relationship between the number of outbreaks and rabbit production per country (a) differentiating the two periods, before and after 2010, (b) when the periods are not distinguished, and (c) relating the number of outbreaks with the rabbit production of the years before RHDV emerged (1961–1985). Fitted regressions are shown with 95% confidence limits.

On considering domestic rabbit production for each country and the number of RHDV/RHDV2 outbreaks reported, we found that the number of reported outbreaks did not differ significantly between the two periods, before and after 2010 (*F* = 0.16, *p*=0.692; Figure 1a). Rabbit production in each country significantly influenced the number of outbreaks reported: there was a positive increasing relationship (*F* = 4.11, *p*=0.047). The interaction between rabbit production and the period was not significant, showing that the same positive relationship occurred in both periods (*F* = 0.03, *p*=0.870). When we removed the period and analysed the relationship between the total number of outbreaks in each country and the average annual rabbit production between 2005 and 2022, we found a positive and significant relationship (*F* = 26.2, *p*  < 0.001, Figure 1b).

**Figure 2:**
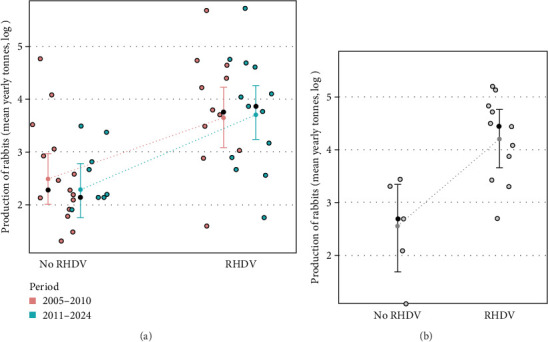
(a) Rabbit production in countries with presence (RHDV) or absence (no RHDV) of reported outbreaks, differentiating the two periods, before and after 2010, when RHDV2 emerged. (b) Rabbit production before the emergence of RHDV (1961–1985) in countries that later had presence or absence of outbreaks. Means (black dots) and standard deviations are shown. RHDV, rabbit haemorrhagic disease viruses.

Moreover, countries reporting RHDV and RHDV2 outbreaks had significantly higher rabbit production (*t* value = 3.14, *p*=0.003) and this effect was independent of the time period (*t* value = 0.63, *p*=0.529) and the interaction term was non-significant (*t* value = 0.60, *p*=0.550) showing that in both periods countries which produced more rabbits reported more outbreaks (Figure 2). These results reject the null hypothesis that there is no relationship between rabbit production and RHDV/RHDV2 outbreaks.

Finally, the total number of outbreaks (RHDV and RHDV2) reported in each country were significantly and positively related to rabbit production before the emergence of RHDV (*F* = 18.50, *p*  < 0.001, Figure 1c); and rabbit production before the emergence of RHDV was significantly higher in countries that later experienced outbreaks of RHDV (*F* = 13.30, *p*=0.003, Figure 2b). This suggests that countries producing more rabbit meat or with more trade in rabbit products had a greater risk of RHDV being introduced.

### 3.2. Risk of Transfer of New Viral Diseases to Native Lagomorphs

**Figure 3:**
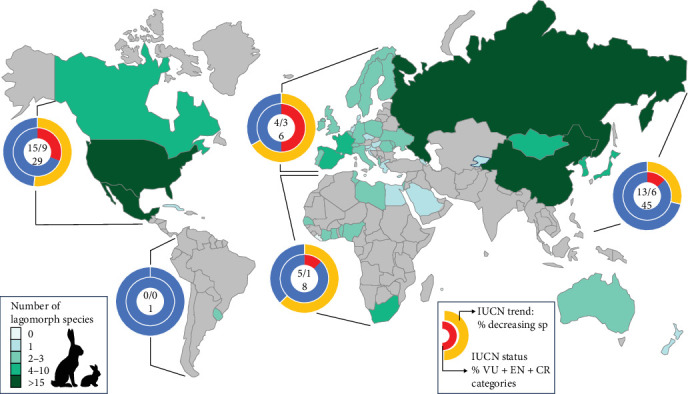
World map of the total number of lagomorph species (native and non-native) in countries that reported outbreaks according to the legend on the left. For each continent, the circles classify the native lagomorph species with the IUCN categories of trend (% of decreasing species, in orange) and conservation status (% of the three most endangered categories, vulnerable, endangered and critically endangered, in red), according to the legend on the right. Inside circles we show on the top the raw number of native species with decreasing trends/the raw number of native species in IUCN most endangered categories, and on the bottom, the total number of native lagomorph species in the countries with outbreaks of each continent. IUCN, International Union for Nature Conservation.

We recorded a total of 83 species of native lagomorphs in the 56 countries having detected or declared suspicious RHDV or RHDV2 outbreaks. The average number of species per country was 3.92 and it ranged from 1 to 32 (see Supporting Information Table S3). Almost a quarter of these species were categorised by the IUCN as vulnerable, endangered or critically endangered (20 species) and more than 40% had decreasing populations (35 species) (Figure 3, Supporting Information Table S4).

**Figure 4:**
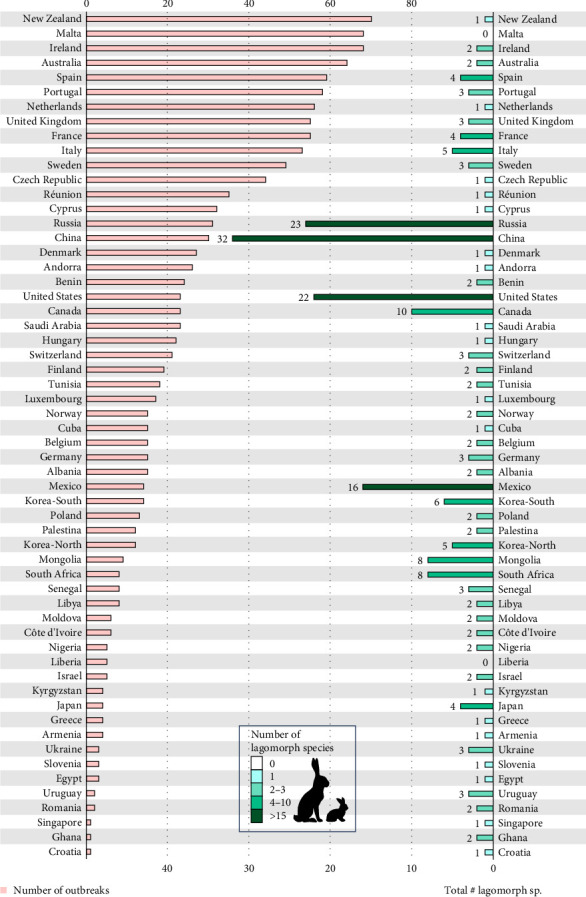
Outbreaks reported by each country and the number of total wild lagomorph species in those countries, including native and non-native species.

The mean number of reported outbreaks per country was 22.4, ranging from one in countries such as Croatia, Ghana or Singapore to more than 65 in countries such as in Ireland (68), Malta (68) or New Zealand (70) (Figure 4). Countries with the highest numbers of native lagomorph species, such as Russia and China, have reported only average number of outbreaks of RHDV/RHDV2 to WOAH since 2005 (31 in Russia, 30 in China). There has been an increased rate of reporting of outbreaks from the United States and Mexico, where RHDV2 has recently spread (23 in the United States and 14 in Mexico).

### 3.3. Trends in Reporting

**Figure 5:**
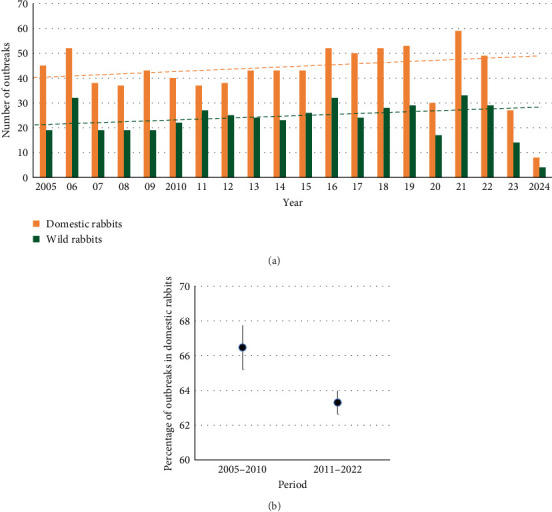
(a) Number of outbreaks in domestic and wild rabbits by year, and (b) percentage of outbreaks in domestic vs wild rabbits. In (a) dashed line is the linear fit for the years 2005–2022, for domestic (orange) and wild rabbits (green). In (b) mean and standard deviations are shown.

Most of the outbreaks reported worldwide were in domestic rabbits (64.4% on average) (Figure 5). The trend in the number of outbreaks increased through time for both domestic and wild rabbits, although it only approached significance for outbreaks in wild rabbits (domestic: X^2^ = 2.37, *p*=0.124; wild: X^2^ = 3.56, *p*=0.059, Figure 5a). As a percentage of all outbreaks, reports of RHDV/RHDV2 in domestic rabbits were significantly lower after the emergence of RHDV2 than before (X^2^ = 4.18, *p*=0.041, Figure 5b), suggesting that there was increasing interest in outbreaks affecting new lagomorph species.

## 4. Discussion

### 4.1. RHDV/RHDV2 Outbreaks Are Globally Correlated With Rabbit Production and Trade

From published data on rabbit production from FAO and reporting of RHDV/RHDV2 outbreaks by WAHIS we found that outbreaks reported by each country are significantly correlated with rabbit meat production. Rabbit production is significantly higher in countries having outbreaks, and the number of outbreaks reported is significantly related to the amount of rabbit meat produced in the countries where outbreaks occur (Figures 1 and 2).

These statistically significant results support the idea, based on published observations, that RHDV and RHDV2 have been spread by trade in live rabbits, rabbit fur and frozen rabbit meat [11–13], and that production and trade in domestic rabbit products facilitate the international spread of RHD. The resilience of virions to harsh environmental conditions also means that indirect fomite transfer between rabbitries is highly likely.

Nonetheless, the results are not conclusive, and other explanations of the correlation cannot be excluded at this stage. It is possible, for example, that in countries with highly developed rabbit meat industries, producers are more aware of disease risks and new diseases are readily reported. Nonetheless, this tends to confirm the argument that the risk of epidemics is increased because of activities associated with domestic rabbit production.

Because there are marked differences between countries in terms of disease reporting and rabbit meat production, our analysis is only a first step and many additional variables would need to be considered for any deeper analysis of the available data, probably requiring more details of each country, such as per capita gross domestic product, traditional foods and climate, to name only a few. Sun et al. [56] describe several environmental and social variables associated with the worldwide distribution of RHDV2 in wild lagomorphs that would be worth considering too.

Nonetheless, a highly detailed analysis is beyond the scope of this initial study and in the absence of further work, we must regard the underlying significant correlation between disease spread and rabbit production as indicating a significant risk factor that would be unwise to ignore.

### 4.2. Risk of Virus Transfer Between Lagomorph Species

We know that, thus far, RHDV2 has spread from domestic rabbits into at least 17 different species and subspecies of lagomorphs in eight different countries (Table 1). We also show that in the 56 countries having detected or declared suspicious RHDV or RHDV2 outbreaks, there are 83 different species of native lagomorphs. China has the highest number of native species closely followed by Russia, then the USA and Mexico. South Africa also has a substantial number of native lagomorphs (Figure 4). Of those lagomorph species, almost a quarter were categorised by the IUCN as vulnerable, endangered or critically endangered (20 species) and more than 40% were decreasing in abundance (35 species) (Figure 3, Supporting Information Table S4).

This not only begins to identify those countries in which the risk of disease spreading from domestic rabbits into native lagomorphs may be high, but it also provides an indication of the potential for new diseases to emerge among domestic rabbits where they are kept close to wild rabbits and hares. China, which produces most rabbit meat internationally also has the most native lagomorph species but outbreaks of RHDV2 were first registered only in 2020 [57]. As the varying hare (*L. timidus*) occurs in China and the Irish subspecies (*L. t. hibernicus*) is known to be susceptible to RHDV2 [34], it is likely that RHDV2 will be reported from wild Chinese lagomorphs in the future.

Importantly, it has been shown that all circulating RHDV2 viruses come from recombination events between different lagoviruses [7]. The original RHDV2 strain identified in France in 2010 resulted from recombination between a non-pathogenic virus (GI.3), donating the genome sequence corresponding to the non-structural genes, and an unknown lagovirus donating that corresponding to the RHDV2 structural proteins (VP1 and VP2). Therefore, strictly, the only genomic sequence known from the original RHDV2 virus is that encoding the structural proteins. Subsequently, the novel RHDV2 virus expanded throughout the rabbit range worldwide, and numerous new recombinant strains have been reported across the globe [36, 58–60]. These recombinants have included almost all possible combinations of lagoviruses: non-pathogenic GI.3 and GI.4, pathogenic GI.1 and even European Brown Hare Syndrome Virus (EBHSV or GII.1). In hares in Germany [61], EBHSV donated the non-structural protein genes, and RHDV2 donated the structural genes. This means that recombination has played a central role in the evolution and dissemination of RHDV2 (pathogenic and with an expanded host-range).

Indeed, the lagovirus family keeps growing due to the detection of previously unnoticed non-pathogenic viruses circulating in different parts of the world. These include the recently reported non-pathogenic lagovirus from Chile (GI.4f variant) [62], a lagovirus strain found in a metagenomics analysis performed in China [63], or RCV viruses detected in Italy from samples collected over the last two decades, including genetically different RCV strains (i.e., proposed new genotypes GI.5 and GI.6) [64]. This implies that there are many different lagoviruses circulating worldwide, which could recombine and give rise to new pathogenic strains.

### 4.3. Other Rabbit Diseases That Might be Similarly Spread Worldwide

Although RHD is the best documented disease of European rabbits because of its relatively recent emergence and spread, other disease-causing viruses are also likely to be spread through trade in live rabbits, their meat and other products. These include the well-known myxoma virus (MYXV), rHEV and lapine rotaviruses and astroviruses which cause disease in rabbitries [65–70]. The latter are generally associated with a problem referred to as the rabbit enteritis complex (REC) [71, 72 ], in young rabbits. New non-pathogenic anelloviruses in Iberian hares have also been recently identified [73].

At least two different lapine rotavirus strains have been shown to cause acute gastroenteritis in human infants [74, 75, 76]. However, currently, none of these lesser-known viruses are routinely tested for in diagnostic laboratories. Recent viral metagenomics studies have detected the presence of rotaviruses or astroviruses in wild rabbits from France [77], Chile [62] and rabbit ectoparasites from Australia [78].

Five herpesviruses have also been identified in leporids: Leporid herpesvirus types 1–5 (LeHV-1 to LeHV-5) [65, 66]. LeHV-1 and LeHV-3 have been isolated from Eastern cottontails *Sylvilagus floridanus*, while LeHV-2 and LeHV-4 infect European rabbits, and the recently reported LeHV-5 was isolated from Iberian hares *Lepus granatensis*. The different herpesviruses have diverse effects on the European rabbit, with LeHV-1, LeHV-2 and LeHV-3 being either non-infectious or passing unnoticed, while LeHV-4 is highly pathogenic, causing fatal infections. Reports to date of LeHV-4 infection are limited to commercial rabbitries and pet domestic rabbits in Alaska and Canada [79, 80]. LeHV-4 has been classified as a member of the Alpha herpesvirus subfamily, while the rest of leporid herpesvirus belong to the Gamma herpesvirus subfamily.

Because these viruses are transmitted in different ways, with MYXV being transmitted mostly by biting insects and RHDV and rHEV usually by direct contact between rabbits or fomites, there are differences in the involvement of wild and domestic rabbits in the spread of each virus. For example, MYXV mostly circulates in wild rabbit populations but may occasionally spread to domestic rabbits, whereas RHDV is highly infective and can be transmitted by carrion flies [81], more regularly spreading in both domestic and wild rabbits at the same time.

In Europe, an ‘amyxomatous' or respiratory form of myxomatosis, which evolved in farmed rabbits is common in domestic rabbitries [82] and is mostly transmitted by close contact rather than insect vectors. Although it is occasionally seen in wild rabbits [83] this cannot explain the presence of amyxomatous myxomatosis in countries such as Egypt and mainland Greece, where there are no wild rabbits. Kritas et al. [84] state that outbreaks of acute myxomatosis on two widely separated Greek rabbit farms followed the arrival of new breeding stock from the same supplier. Again, such examples implicate trade in rabbits as a major cause of disease spread.

Stern et al. [85] remark that in California MSW myxomatosis from brush rabbits (*Sylvilagus bachmani*) frequently infects domestic rabbits, although in all reported cases the domestic rabbits had been allowed to spend time outdoors despite known disease risk. Such reports show the ongoing potential for viruses to spread between domestic and wild European rabbits or between different lagomorph species in either direction.

Although MYXV occasionally caused infections of European hares (*L. europaeus*) in the wild [86], the spread and persistence of myxoma virus in Iberian hares (*L. granatensis*) since 2018 is more alarming and should be regarded as a host species jump [87, 88]. Thousands of hares have been killed by this new hare myxoma virus (ha-MYXV), which is a recombinant strain harbouring a DNA insertion from an unknown poxvirus encoding several genes. Included among them is a new gene (M159), apparently involved in its expanded host range [89]. The new ha-MYXV has also been shown to cause fatal infection in both domestic and wild populations of European rabbit [90], and recent reports from Spain, Netherlands and Germany indicate this new ha-MYXV can also infect European hares (*L. europaeus*) [91, 92, 93].

In developing countries, where rabbit production is encouraged in small villages and rural communities to alleviate poverty and improve protein intake, domestic rabbits are kept for small-scale household production and often in areas where native lagomorphs are present [94]. Rabbits are usually fed on kitchen waste while their meat is mostly consumed by the family, although surplus animals may be sold [95]. Nevertheless, because RHDV can survive for prolonged periods and resist environmental degradation, there is high potential for transfer of viral diseases where herbage is collected from areas inhabited by wild lagomorphs to feed caged rabbits and faeces and soiled bedding from domestic rabbits is spread on fields as fertiliser. Rabbit production in those countries rarely develops into a major tightly controlled industry because costs rise when rabbit feed and vaccines need to be purchased, and rabbit keeping is no longer a simple family enterprise [96]. Consequently, there is an ever-present risk of virus transfer between wild and domestic lagomorphs.

### 4.4. Implications of Virus Emergence for Domestic Rabbit Production

In Europe, where consumption of rabbit meat is traditional, countries such as Spain, France, Italy and Belgium developed large rabbit producing industries. By the late 1980s, over 600,000 tonnes of rabbit meat were being produced annually in Europe representing 87% of estimated world production [97, 98].

Rabbit production and trade of rabbit products have since expanded through Asia, Africa and the Americas and now China produces over 60% of rabbit meat globally although it is not the highest exporter. Even in India a rabbit meat and fur industry is under development, although there is not yet a significant export industry for rabbit products [99, 100].

Associated with the spread of RHDV, between 1986 and 1991, rabbit meat production in Europe fell by over 30% and has since continued to decline ([101] and FAO data). Lower production was briefly offset by increased production in Asia, Africa and the Americas, but production has since fallen in the two last mentioned regions leaving Asia as the biggest producer of rabbit meat.

The spread of RHD also led to a crippling of projects aimed at using domestic rabbits to alleviate poverty. For example, in Mexico, before an outbreak of RHD in 1989, 70% of villagers in the small rural community of Xocotlan kept rabbits, but rabbit-keeping declined to 45% after the outbreak [102]. Many people who lost rabbit breeding stock due to RHD lost interest in rabbit keeping or could not afford the extra costs of animal hygiene measures and vaccination and did not replace the rabbits that died. With fewer people producing rabbits in Mexico, most of the rabbit meat produced is now consumed within the country, explaining why the share of the world rabbit meat market from the Americas fell from about 3% in 1991 to only 1% by 2018. Now that RHDV2 is endemic in wild lagomorphs in North America [103], vaccination and other costly animal hygiene measures have become essential for maintaining rabbit production. This means that rabbit production will be less profitable, and further contraction of cuniculture in North America should be anticipated.

Unfortunately, even with the growing evidence that known diseases can spread into a wide range of lagomorph species, it is not widely appreciated by domestic rabbit owners that there is a major risk of transfer of viruses where their rabbits are kept close to native lagomorphs. This is not simply a problem in small villages reliant on rabbit meat. Shapiro et al. [104] found that over 10% of owners of pet domestic rabbits surveyed in the USA frequently allowed their rabbits to roam in areas occupied by wild lagomorphs. Shapiro et al. [105] further found that the responsibilities of government agencies were poorly defined when it came to managing RHDV2 in pet rabbits and wild species. This was partly because domestic rabbits are not treated like other domestic livestock, and there was limited knowledge of the size and composition of the domestic rabbit industry or nearby wild lagomorph populations.

### 4.5. Implications for the Biological Control for Invasive Rabbits

Associated with research on the use of viruses and their genetic modification for the biological control of wild rabbits in the 1990s and early 2000s [106–109], regulations controlling research on biological control agents are now very strict. As in the case of the cyprinid herpesvirus-3, advocated for the control of introduced carp (*Cyprinus carpio*) in Australian rivers [110], assessment of new and high-risk viruses as possible biological control agents must be done carefully in quarantine, in high level (BSL-3 and BSL-4) physical containment facilities before seeking permission to release.

Nonetheless, the rapid international spread of RHDV2 makes it clear that the virus should not be manipulated genetically to further bolster its effectiveness as a biological control agent of rabbits in Australia or New Zealand. The variant of RHDV2 initially detected in Australia by chance [111] closely resembled one from Portugal [112] raising the possibility of rapid transfer on clothing or footwear of air passengers. Furthermore, RHDV2 arrived in Australia and New Zealand independently rather than by transfer between nearby countries. This indicates the near impossibility of confining such viruses [113] and potentially, any genetically modified viruses could spread into native lagomorph populations in other continents, again via domestic rabbits and people associated with pet and meat producing industries.

Despite these risks, however, there is a need to maintain a high level of biological control of rabbits in Australia because rabbits occur widely in uninhabited arid inland areas. Not only are human resources lacking but chemical or mechanical control of rabbits is uneconomical because costs are high and even in livestock producing areas the monetary returns from extra livestock production following removal of rabbits are uncertain. From a conservation perspective, rabbits at population densities of only one rabbit to 20 ha can remove all new germinants and seedlings of highly palatable shrubs such as *Acacia* spp., which are a major component of Australian arid-zone vegetation [114].

Further work on the use of viruses for the biological control of rabbits in countries like Australia and New Zealand will need to be done very cautiously and must include safeguards to ensure that any new or modified agent can be confined and not spread internationally.

### 4.6. Rabbit Diseases, Host Specificity and Evolution

Although the spread of RHDV2 into a wider range of lagomorphs is a set-back in terms of conservation, its spread into 15 new species on three continents provides a unique opportunity to compare the susceptibility of different lagomorph species to the same virus. Further study would provide insights into the coevolution of viruses and their new hosts. Newly emergent diseases in rabbits are of particular interest because, unlike other domestic animals such as cattle or the human population, it is possible to study virus-host coevolution in wild lagomorph populations unaffected by countermeasures such as vaccination.

The rapid evolution of resistance to MYXV in European rabbits has been measured in real time because wild European rabbits have a generation time of only 1 year [115] and, with modern genetic sequencing techniques, nucleotide changes in both the virus and the host can be readily monitored [17, 116, 117].

Interestingly, although eastern cottontails (*S. floridanus*) can be experimentally infected with RHDV2 [40] it does not spread in their natural wild populations in the USA (or introduced populations in Italy) as readily as it spreads among desert cottontails (*Sylvilagus audubonii*) or black-tailed jackrabbits (*Lepus californicus*). This raises many questions about the factors that make wild eastern cottontails less susceptible. Is it the result of cottontail species differences or differences in climate within each species' distribution, for example?

Understanding more about factors that influence disease susceptibility and spread could be helpful for understanding other disease-causing viruses, including those which affect humans.

## 5. Conclusions

From our considerations of recently emerged viral diseases in European rabbits, we conclude that irrespective of the origin of the viruses, there is great potential for their spread throughout the world because domestic rabbits are present in almost every country, commonly for meat and fur production, but also as laboratory animals used in research institutions or as household pets.

Unlike the major livestock industries, trade in rabbit products is often unrestricted, and there is a lack of information about rabbits kept on small family farms or as pets. Many local authorities are unclear about their responsibilities for managing domestic rabbits and are ill-prepared for handling disease outbreaks. Biosecurity measures are not yet widely acquired as in other sectors (e.g., poultry due to the spread of avian influenza and pigs following the appearance of African swine fever or classical swine fever). Instead such considerations are hampered by the low consideration that health authorities have of rabbit viral diseases despite their high contagiousness and severity (i.e., rabbit viral diseases have not been included in the diseases of community interest (Reg. EC 2016/429), leaving the discretion of notification and adoption of containment measures to each country). This situation in turn provides a superhighway for highly contagious viruses such as RHDV2 to rapidly spread worldwide. The consequences of the spread of diseases, and RHDV/RHDV2 especially, have seen shifts in world rabbit meat production at both the national level and the level of family farms due to the added cost of disease control through vaccination and added farm hygiene practices. A stricter international biosecurity approach to rabbit production and management is clearly required, like that commonly suggested for other species of zootechnical interest.

Apart from Australia and New Zealand, where there are no native lagomorphs, in those countries where RHDV2 has recently been recorded, we show that there are on average about four native lagomorph species potentially at risk although the distribution is highly skewed (range 1–31). The worldwide distribution of domestic rabbits, significant trade in rabbit products, and opportunities for transfer of several known diseases between domestic rabbits and many different species of wild lagomorphs, means we should be alert for future two-way traffic of rabbit diseases, into domestic rabbits from wild species or from domestic rabbits into wild species.

Because rabbit production is economically important for countries and human societies, we propose some measures (Box 1) that could help to prevent further virus spread. Experience with RHDV2 confirms that the spread of viruses that affect lagomorphs constitutes an ongoing risk; it is not simply a theoretical possibility.

## Figures and Tables

**Box 1:**
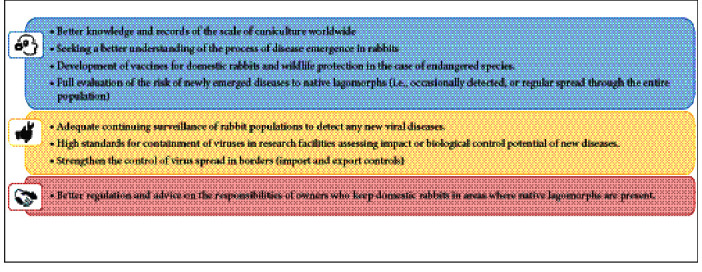
Measures to prevent further spread of virus diseases in the future.

**Table 1:** Verified Leporid host species of RHDV and RHDV2.

Species	RHDV (GI.1)	RHDV2 (GI.2)	First detection of RHDV2	References RHDV2
*Oryctolagus cuniculus*	+	+	France, 2010	[4, 20]
*Lepus capensis mediterraneus*		+	Sardinia (Italy), 2011	[28]
*Lepus corsicanus*		+	Sicily (Italy), 2012	[29]
*Lepus europaeus*		+	Lombardy (Italy), 2012	[30–32]
*Lepus timidus*		+	Sweden, 2016	[33–35]
*Lepus granatensis*	+	+	Spain, 2020	[36]
*Lepus alleni*		+	California (USA), 2020–2021	[37]
*Lepus californicus*		+	California (USA), 2020–2021	[37–39]
*Sylvilagus audubonii*		+	California (USA), 2020–2021	[37–39]
*Sylvilagus nutalli*		+	California (USA), 2020–2021	[37]
*Sylvilagus floridanus*		+	(USA), 2021 (experimental)	[40]
*Sylvilagus bachmani*		+	California (USA), 2021	[41]
*Sylvilagus bachmani riparius*		+	California (USA), 2022	[41]
*Brachylagus idahoensis*		+	Nevada (USA), 2022	[42]
*Lepus capensis*		+	South Africa, 2022	[22]
*Lepus saxatilis*		+	South Africa, 2022	[22]
*Pronolagus* spp.		+	South Africa, 2022	[22]

## Data Availability

The original contributions presented in the study are included in the Supporting Information.

## Supporting Information

Supporting Information Table S1 is an Excel file that contains data used in the article and is separated in four spreadsheets: Table SI_1_WAHIS contains the data from WOAH; Table SI_2_FAOSAT contains data from FAO; Table SI_3_Lagomorph_byCountry contains the list of lagomorph species by country and their trend and status following the IUCN; and Table SI_4_Lagomorph_bySp, contains the 85 native lagomorph species with their trend and status following IUCN.
